# Prebiopsy bpMRI and hematological parameter-based risk scoring model for predicting outcomes in biopsy-naive men with PSA 4–20 ng/mL

**DOI:** 10.1038/s41598-022-26242-7

**Published:** 2022-12-19

**Authors:** Yuxin Zheng, Wang Li, Yang Zhang, Chi Zhang, Junqi Wang, Peng Ge

**Affiliations:** Department of Urology, The Affiliated Hospital of Xuzhou Medical University, Xuzhou, China

**Keywords:** Prostate, Cancer imaging, Cancer models, Cancer screening, Prostate cancer, Diagnostic markers, Risk factors

## Abstract

Excessive prostate biopsy is a common problem for clinicians. Although some hematological and bi-parametric magnetic resonance imaging (bpMRI) parameters might help increase the rate of positive prostate biopsies, there is a lack of studies on whether their combination can further improve clinical detection efficiency. We retrospectively enrolled 394 patients with PSA levels of 4–20 ng/mL who underwent prebiopsy bpMRI during 2010–2021. Based on bpMRI and hematological indicators, six models and a nomogram were constructed to predict the outcomes of biopsy. Furthermore, we constructed and evaluated a risk scoring model based on the nomogram. Age, prostate-specific antigen (PSA) density (PSAD), systemic immune-inflammation index, cystatin C level, and the Prostate Imaging Reporting and Data System (PI-RADS) v2.1 score were significant predictors of prostate cancer (PCa) on multivariable logistic regression analyses (*P* < 0.05) and the five parameters were used to construct the XYFY nomogram. The area under the receiver operating characteristic (ROC) curve (AUC) of the nomogram was 0.916. Based on the nomogram, a risk scoring model (XYFY risk model) was constructed and then we divided the patients into low-(XYFY score: < 95), medium-(XYFY score: 95–150), and, high-risk (XYFY score: > 150) groups. The predictive values for diagnosis of PCa and clinically-significant PCa among the three risk groups were 3.0%(6/201), 41.8%(51/122), 91.5%(65/71); 0.5%(1/201), 19.7%(24/122), 60.6%(43/71), respectively. In conclusion, in this study, we used hematological and bpMRI parameters to establish and internally validate a XYFY risk scoring model for predicting the biopsy outcomes for patients with PSA levels of 4–20 ng/mL and this risk model would support clinical decision-making and reduce excessive biopsies.

## Introduction

The incidence and mortality of prostate cancer (PCa) have been increasing rapidly worldwide^1^, and China is no exception^2^. Prostate biopsy is a common method for diagnosing PCa based on measurements of prostate-specific antigen (PSA) level, imaging examination, and digital rectal examination (DRE). However, PSA is not a PCa-specific marker and the detection rate of PCa with PSA levels of 4–20 ng/mL is 25% or less^3^. Meanwhile, DRE, which is highly subjective and shows low consistency, cannot easily detect tumors in the transition zone or smaller than 0.5 cm. Moreover, cancerous lesions may show a soft texture, which can lead to higher false-negative results^4^.

In the guidelines, prebiopsy magnetic resonance imaging (MRI) is supported by level B evidence or a strong recommendation^5,6^. In 2019, the European Society of Urogenital Radiology (ESUR) and other organizations released the Prostate Imaging Reporting and Data System version 2.1 (PI-RADS v2.1) for standardized prostate reporting^7^. Bi-parametric MRI (bpMRI), proposed in PI-RADS v2.1, only includes T2-weighted imaging (T2WI) and diffusion-weighted imaging (DWI), which can simplify the process of prostate MRI scanning. Moreover, some studies have shown that the diagnostic accuracy and performance of bpMRI are comparable to those of multi-parameter MRI (mpMRI)^8–10^.

Systemic inflammation has been demonstrated to be associated with carcinogenesis and cancer progression^11–13^. Among inflammatory markers, the systemic immune-inflammation index (SII) has been suggested to be a more powerful predictor of tumors than the neutrophil-to-lymphocyte ratio (NLR) and platelet-to-lymphocyte ratio (PLR)^14,15^. Other hematological indicators, such as the red blood cell distribution width (RDW) and cystatin C (CysC) level, are also related to the occurrence and progression of tumors^16,17^. Nevertheless, studies on the use of these indicators as predictors of PCa are currently lacking.

Therefore, this study utilized hematological and bpMRI indicators to establish a risk scoring model for predicting the outcomes in biopsy-naive men with PSA levels of 4–20 ng/mL and thereby support clinical decision-making for prostate biopsy.

## Patients and methods

### Study population

This was a retrospective, single-institution study approved by the ethical committee of the Affiliated Hospital of Xuzhou Medical University (XYFY2022-KL192-01). Patients who had elevated PSA levels of 4–20 ng/mL and subsequently underwent prostate biopsy at our institution between March 2010 and April 2021 were included. Inclusion criteria: (1) Patients underwent transrectal ultrasonography-guided systematic prostate biopsy and all the biopsies were done at our hospital; (2) Patients underwent prebiopsy MRI scans and the T2WI and DWI data could be obtained; (3) Comprehensive clinicopathological data were available. Exclusion criteria: (1) Acute inflammation diseases, such as acute bacterial prostatitis; (2) Patient underwent prior prostate surgery or biopsy; (3) Patients had indwelling urethral catheters within 48 h of PSA detection; (4) Patients had prior hematological diseases. For systematic prostate biopsy, 10 + X cores scheme was mostly used^18^. The mean value was 10.1 cores. Totally, 394 patients were enrolled in this study. All methods were performed in accordance with the relevant guidelines.

### Imaging evaluation

Patients were scanned with MRI scanners with a multichannel-body-surface coil. The MRI sequences at least included T2WI (axial, sagittal and/or coronal) and DWI. All MRI images were re-reviewed by the same person (author Yuxin Zheng) under the guidance of a dedicated radiologist with more than 10 years of MRI experience. The PI-RADS v2.1 guidelines^7^ were utilized in this study. The reviewer was blinded to histopathologic diagnosis and previous MRI reports.

### Clinical parameter collection

The patients’ clinicopathological data, such as biopsy outcomes, Gleason score (GS), PI-RADS v2.1 score, height, weight, age, PSA, total PSA (tPSA), free PSA (fPSA), free/total PSA (f/tPSA), albumin, globulin, RDW, CysC, red blood cell (RBC) count, hemoglobin (Hb), alkaline phosphatase (ALP), and fibrinogen levels, were obtained from their medical records.

Prostate volume (PV) was measured using the exact prolate ellipsoid formula: PV (mL)^7^ = transverse diameter (cm) × anteroposterior diameter (cm) × longitudinal diameter (cm) × 0.52. PSA density (PSAD) (ng/mL) was calculated as the ratio of tPSA to PV^19^. Body mass index (BMI) (kg/m^2^) was calculated using height and weight measurements. Albumin-to-globulin ratio (AGR) was calculated as the ratio of albumin to globulin levels. SII was calculated as platelet count × neutrophil count/lymphocyte count^20^. Additionally, we defined PI-RADS 1 and 2 scores as score < 3. Clinically insignificant PCa (cisPCa) was defined as International Society for Urological Pathology (ISUP) grade 1 (GS ≤ 6), otherwise clinically significant PCa (csPCa)^6^.

### Statistical analysis

Descriptive statistics relied on tests of medians [interquartile range (IQR)] and proportions (rates), Mann–Whitney U test or chi-squared test. For risk model construction, continuous variables were transformed into categorical variables based on cut-off values. Univariable and multivariable logistic regression were used to examine factors associated with Pca. The nomogram was constructed on the basis of independent predictors. The effectiveness and precision of the nomogram were assessed using area under the receiver operating characteristic (ROC) curve (AUC) measurements, calibration plots, and decision-curve analysis (DCA). Finally, a web-based interactive tool was developed to predict the PCa. A two-sided *P* < 0.050 was considered to be statistically significant. Analyses were performed using Statistical Product and Service Solutions software (SPSS, version 26.0) and R software (version 4.1.3).

### Ethical approval

This was a retrospective study approved by the affiliated hospital of Xuzhou Medical University's ethics committee (No. XYFY2022-KL192-01) who waived informed consent.

## Results

### Patient characteristics

A total of 394 participants, including 272 (69.04%) biopsy-negative cases and 122 (30.96%) biopsy-positive cases, were enrolled in this study (Table 1). Compared to the negetive group, the positive group had older age, higher tPSA/PSAD/SII/CysC levels, and lower [f/tPSA]/PV/RBC/Hb levels (*P* values < 0.05, Table 1). The median score of PI-RADS v2.1 was 3.0 (Table S1, Supplementary Materials).

**Table 1:** Descriptive characteristics of clinical parameters with negative and positive biopsy.

Variable [median (IQR)]	Negative	Positive	*P* value
	(n = 272)	(n = 122)	
Age (year)	67.00 (61.00–73.00)	72.00 (65.00–77.00)	< 0.001
BMI (kg/m^2^)	24.49 (22.86–26.39)	24.06 (22.49–26.09)	0.357
tPSA (ng/mL)	9.25 (6.94–12.73)	11.04 (7.71–15.45)	0.001
fPSA (ng/mL)	1.20 (0.84–1.69)	1.17 (0.70–1.73)	0.627
f/tPSA	0.13 (0.09–0.18)	0.11 (0.07–0.16)	0.003
PV (mL)	54.24 (38.86–80.88)	36.12 (24.86–49.38)	< 0.001
PSAD	0.17 (0.12–0.23)	0.30 (0.20–0.45)	< 0.001
NLR	1.99 (1.49–2.56)	2.09 (1.55–3.05)	0.082
SII	383.84 (289.49–512.60)	430.85 (321.70–722.33)	0.004
AGR	1.57 (1.42–1.74)	1.57 (1.39–1.76)	0.784
RBC (10^12^/L)	4.63 (4.33–4.91)	4.46 (4.26–4.78)	0.006
Hb (g/L)	142.00 (135.00–151.00)	138.00 (130.00–148.00)	0.002
RDW (%)	12.90 (12.50–13.20)	12.95 (12.50–13.40)	0.147
ALP (U/L)	70.00 (58.25–82.00)	69.00 (56.00–84.00)	0.824
CysC (mg/L)	0.85 (0.77–0.91)	0.97 (0.85–1.07)	< 0.001
FIB (g/L)	2.90 (2.33–3.70)	2.96 (2.42–3.68)	0.678

*IQR* interquartile range, *BMI* body mass index, *PSA* prostate-specific antigen, *tPSA* total PSA, *fPSA* free PSA, *f/tPSA* free/total PSA, *PV* prostate volume, *PSAD* PSA density, *SII* systemic immune-inflammation index, *AGR* albumin to  globulin ratio, *RBC* red blood cell, *Hb* hemoglobin, *ALP* alkaline phosphatase, *CysC* cystatin C, *FIB* fibrinogen.

The RBC count and Hb level were transformed into categorical variables according to the lower limit of normal. Moreover, the cut-off values of SII, f/tPSA, PSAD and CysC obtained using the ROC curve analysis were 550, 0.12, 0.25 ng/mL, and 0.9 mg/L, respectively. On the basis of the cut-off values, these variables were transformed into categorical variables. Next, age was transformed into a categorical variable according to the quartile.

Univariable and multivariable logistic regression analyses for prediction associated with PCa were performed using the categorical variables and PI-RADS v2.1 (Table 2). Eventually, age, SII, CysC level, PI-RADS v2.1 score, and PSAD were found to be independent risk factors for PCa.

**Table 2:** Univariable and multivariable logistic regression analysis of factors associated with the outcomes of biopsy.

Variable [n(%)]	Univariable analysis			Multivariable analysis	
	Negative (n = 272)	Positive (n = 122)	*P* value	OR (95% CI)	*P* value
Age			< 0.001		< 0.05
< 60	50 (18.4)	7 (5.7)			Ref
≥60, <75	174 (64.0)	74 (60.7)		1.61 (0.54–4.75)	0.39
≥75	48 (17.6)	41 (33.6)		4.82 (1.45–16.03)	0.1
f/tPSA (0.12)			0.001		
>0.12	163 (59.9)	51 (41.8)			Ref
≤0.12	109 (40.1)	71 (58.2)		0.96 (0.49–1.91)	0.91
PSAD (0.25)			< 0.001		
<0.25	215 (79.0)	42 (34.4)			Ref
≥0.25	57 (21.0)	80 (65.6)		7.65 (3.77–15.53)	< 0.05
SII (550)			< 0.001		
<550	220 (80.9)	76 (62.3)			Ref
≥550	52 (19.1)	46 (37.7)		2.31 (1.18–4.53)	0.015
RBC (4)			0.015		
>4.00	250 (91.9)	102 (83.6)			Ref
≤4.00	22 (8.1)	20 (16.4)		1.32 (0.37–4.89)	0.68
Hb (120)			0.022		
>120	257 (94.5)	107 (87.7)			Ref
≤120	15 (5.5)	15 (12.3)		0.87 (0.20–3.79)	0.86
CysC (0.9)			< 0.001		
<0.9	196 (72.1)	44 (36.1)			Ref
≥0.9	76 (27.9)	78 (63.9)		3.25 (1.69–6.25)	< 0.05
PI-RADS v2.1			< 0.001		
<3	123 (45.2)	4 (3.3)			Ref
3	107 (39.3)	35 (28.7)		13.63 (4.07–45.63)	< 0.05
4	35 (12.9)	46 (37.7)		56.97 (16.28–199.42)	< 0.05
5	7 (2.6)	37 (30.3)		121.02 (27.45–533.53)	< 0.05

*OR* odds ratio, *PSA* prostate-specific antigen, *f/tPSA* free/total PSA, *PSAD* PSA density, *SII* systemic immune-inflammation index, *RBC* red blood cell, *Hb* hemoglobin, *CysC* cystatin C, *PI-RADS v2.1* Prostate Imaging Reporting and Data System version 2.1, *CI* confidence interval, *ref* reference.

### Construction and assessment of the nomogram

In this study, six models were constructed for predicting PCa: (1) The baseline model: only containing age, SII, and CysC; (2)The PI-RADS model: only containing PI-RADS v2.1 score; (3) The baseline + PSAD model; (4) The PI-RADS + PSAD model; (5) The PI-RADS + PSAD + age model; (6) The XYFY model: baseline + PI-RADS + PSAD.

**Figure 1:**
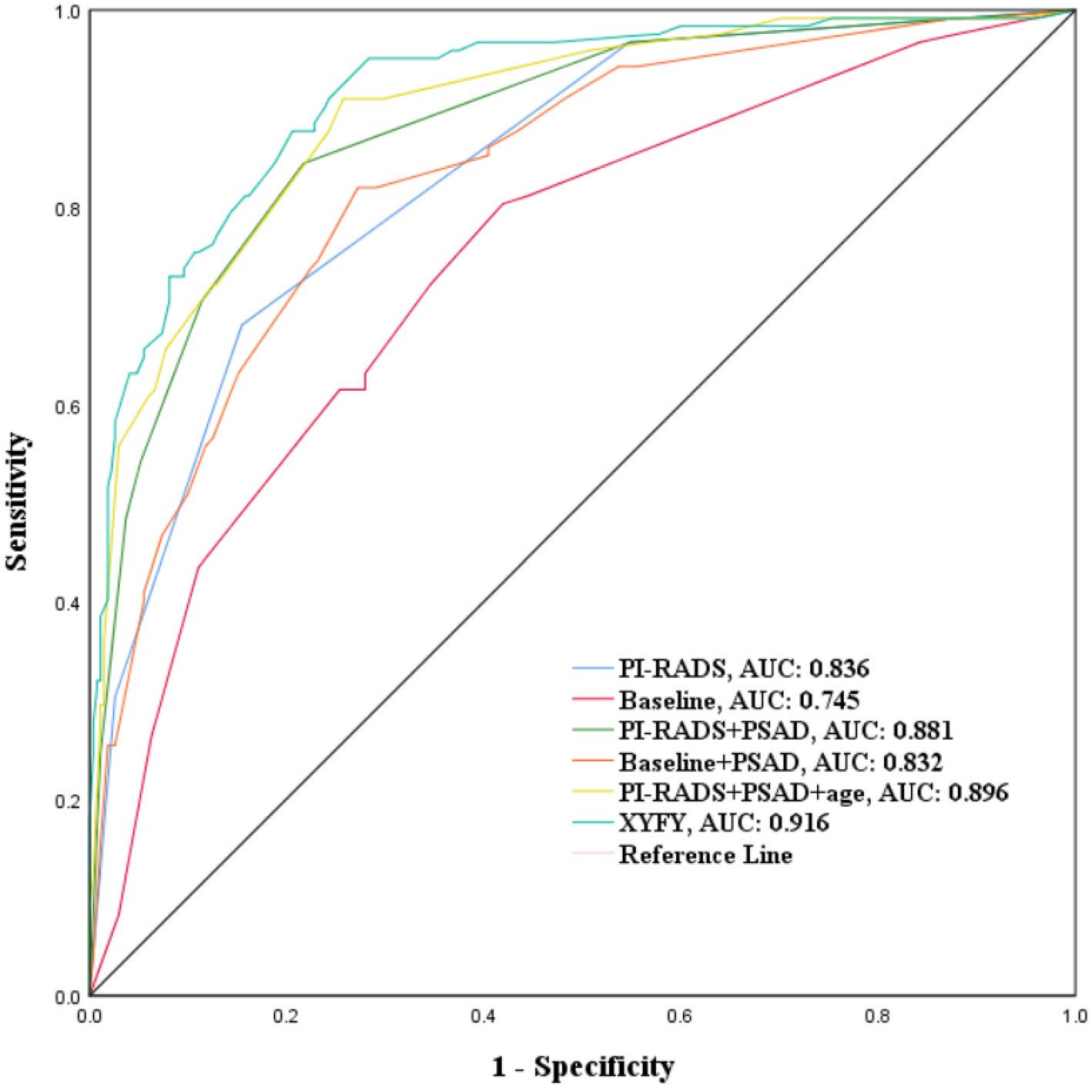
ROC curves comparing PI-RADS with PI-RADS + PSAD model, baseline model (baseline model was constructed on the basis of age, SII, and CysC level), baseline + PSAD model, PI-RADS + PSAD + age model, and XYFY model (XYFY model was constructed on the basis of age, SII, CysC level, PI-RADS v2.1 score, and PSAD) in predicting the outcomes of biopsy. *ROC* receiver operating characteristic; *PI-RADS* Prostate Imaging Reporting and Data System; *PSAD* prostate-specific antigen density.

ROC analyses were performed to evaluate the predictive accuracy of these models (Fig. 1). Figure 1 illustrates that the addition of the PSAD increased the AUC of the baseline model from 0.745 (95% CI 0.692–0.797) to 0.832 (95% CI 0.789–0.875) (*P* < 0.05). The predictive ability of the baseline + PSAD model was equivalent to that of the PI-RADS model (0.832 vs. 0.836, respectively) in predicting the outcomes of biopsy. Moreover, the XYFY model had the highest AUC for discriminating between biopsy-negative and biopsy-positive cases among the six models (0.916, Fig. 1, Table S2, Supplementary Materials).

**Figure 2:**
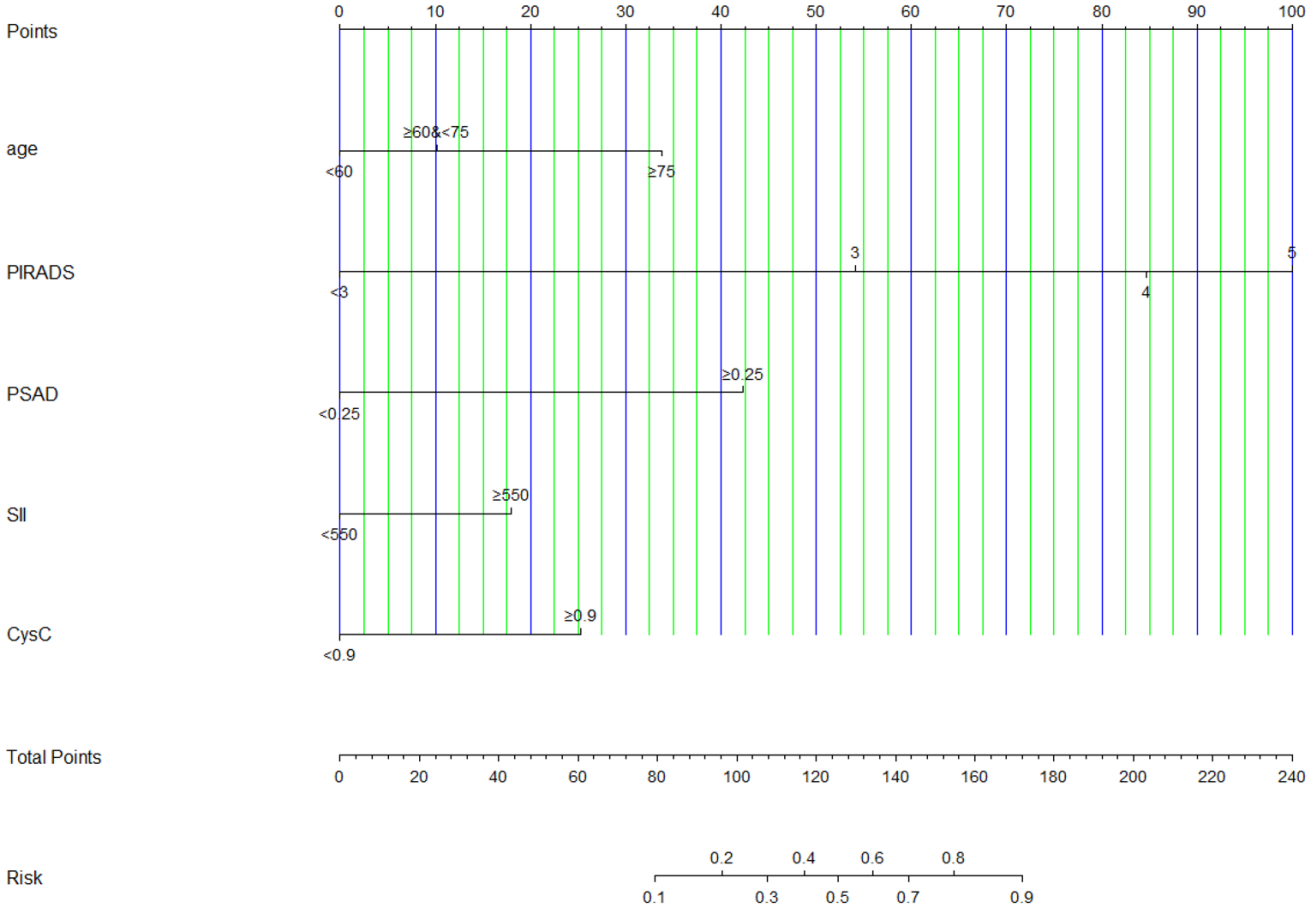
The nomogram for predicting the outcomes of biopsy in biopsy-naive men with PSA 4–20 ng/mL. *PI-RADS* Prostate Imaging Reporting and Data System; *PSAD* prostate-specific antigen density; *SII* systemic immune-inflammation index; *CysC* cystatin C.

Furthermore, the XYFY model, which included age, SII, CysC level, PSAD, and PI-RADS v2.1, was used to construct the nomogram to predict the outcomes of biopsy (Fig. 2). The calibration curve using internally bootstrapped sampling (1000 resamples) showed that the bias-corrected curve was almost identical to the ideal curve, indicating that the nomogram had been calibrated properly (Figure S1, Supplementary Materials). DCA was used to assess the potential clinical benefits of these models and nomograms (Figure S2, Supplementary Materials). Between 0% and the high-risk threshold at approximately 70%, DCA showed that the models and nomogram might generate greater net benefits than all or none, with the XYFY model being more beneficial. The results for internal validation of the XYFY model using the 50 times tenfold cross-validation method yielded high predictive performance and demonstrated good concordance (Accuracy = 0.85; Kappa = 0.63). Finally, we developed a web-based interactive tool based on the final model (https://1riskcalculator.shinyapps.io/dynnomapp/).

### Construction and assessment of the risk scoring model

We established a risk scoring model (XYFY risk model) based on the score calculated by the XYFY nomogram. Patients with a score < 95 were classified as the low-risk group and could be considered as negative for PCa, while those with a score > 150 were categorized as the high-risk group and required a biopsy to confirm the presence of PCa. Finally, patients with a score between 95 and 150 were identified as being in the medium-risk group and had to be actively monitored through follow-up MRI and PSA assessments.Using the XYFY risk model, unnecessary biopsies could be avoided in nearly 49% of patients (195/394), while missing only 3.0% (6/201) of PCa patients. The predictive performance of the XYFY risk model was assessed, and the related results are presented in Table 3 and Table S3. The predictive values for diagnosis of PCa and csPCa among the three risk groups were 3.0% (6/201), 41.8% (51/122), 91.5% (65/71); 0.5% (1/201), 19.7% (24/122), 60.6% (43/71), respectively.

**Table 3:** XYFY risk model for predicting the outcomes of biopsy.

XYFY risk score	Histopathology		Probability of positive (%)
	Negative	Positive	
< 95	195	6	3.0
95–150	71	51	41.8
>150	6	65	91.5

**Figure 3:**
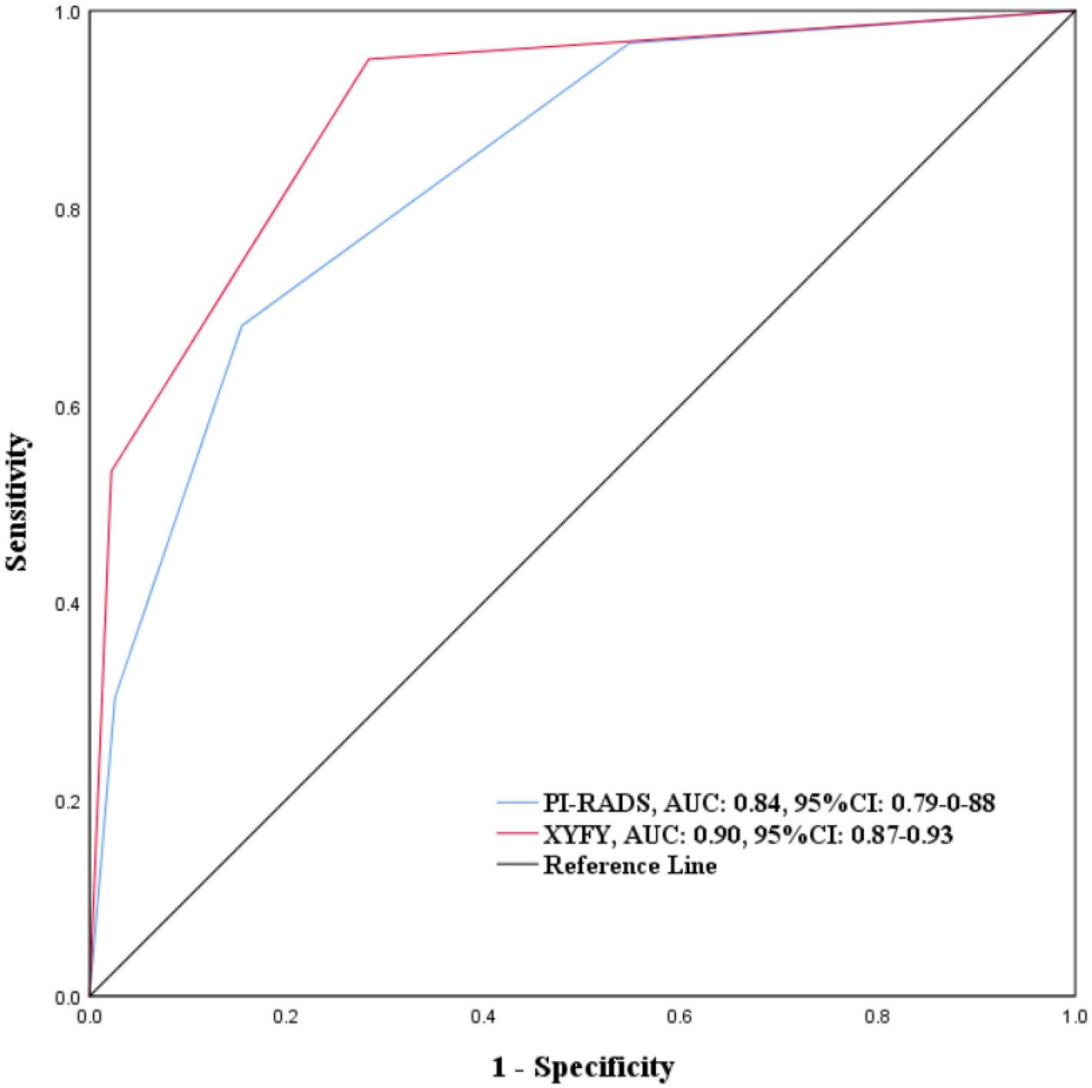
ROC curves for comparing predictive performance of XYFY risk model and PI-RADS. *ROC* receiver operating characteristic; *PI-RADS* Prostate Imaging Reporting and Data System.

Compared to PI-RADS model, our model significantly increased the number of low-risk patients (low risk group vs PI-RADS < 3; 201 vs.127) (Table S3, Supplementary Materials). Moreover, the AUC of the XYFY risk model (AUC = 0.90; 95% CI 0.87–0.93) was significantly better than that of the PI-RADS v2.1 score (AUC = 0.84; 95% CI 0.79–0.88; *P* < 0.001) (Fig. 3). In the suspicious group (PI-RADS 3), the present model reduced the proportion of non-PCa (58.2% vs. 75.4%) and increased the proportion of csPCa (19.7% vs. 12.7%). Compared to the PI-RADS > 3 group, the new high risk group was more precise to diagnose csPCa (60.6% vs. 39%).

**Figure 4:**
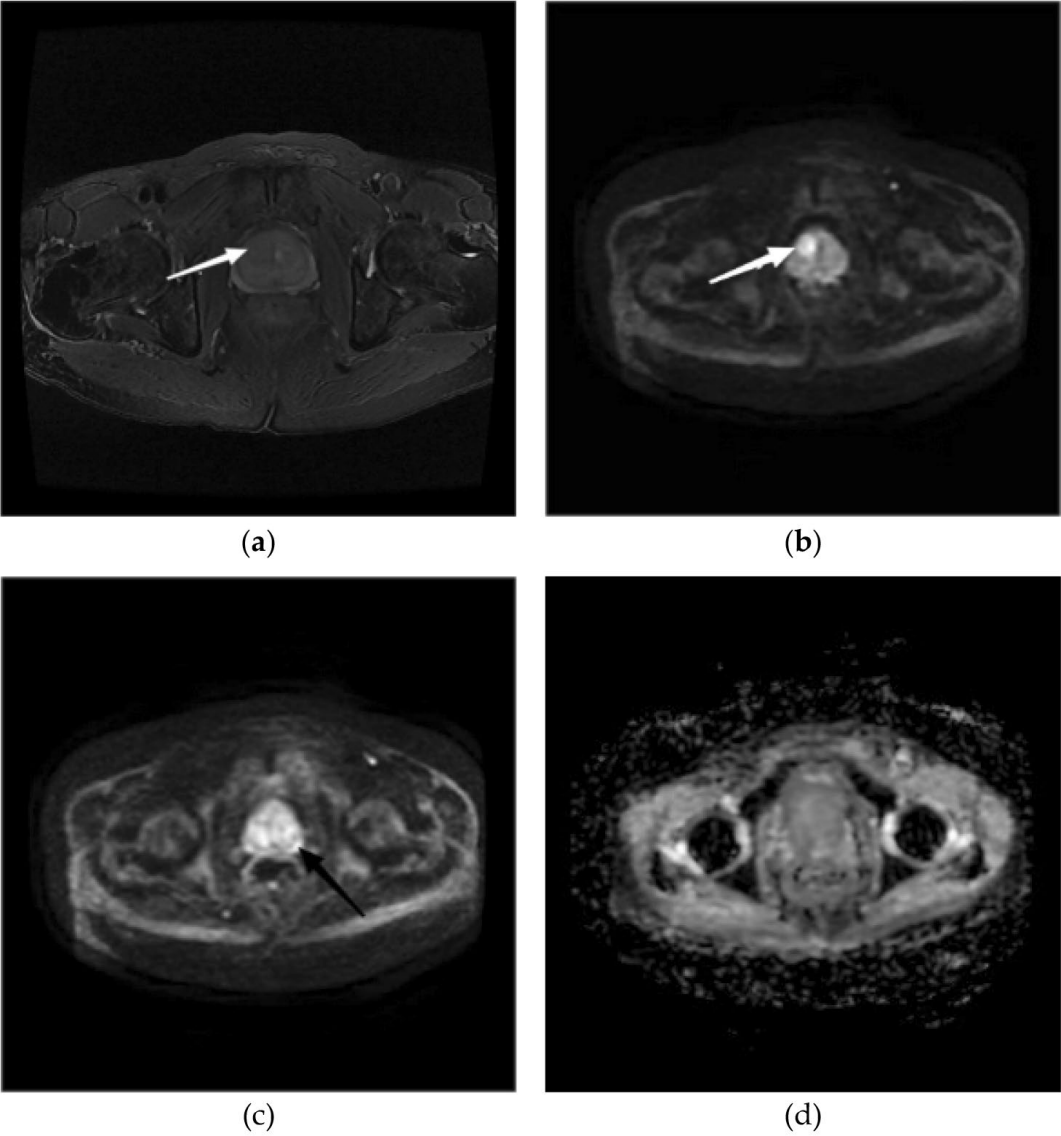
An 80-year-old patient with a PI-RADS v2.1 score of 3 + 3. Transition zone (TZ): (**a**) Axial T2WI shows a heterogeneous signal intensity with obscured margins nodule (white arrow). (**b**) DWI map shows a focal lesion with a markedly hyperintense signal (white arrow) corresponding to the lesion seen in (**a**). T2WI PI-RADS = 3, DWI PI-RADS = 4, PI-RADS assessment category = 4. Peripheral zone (PZ): (**c**) DWI map shows a focal lesion with a markedly hyperintense signal (black arrow) in the right PZ. (**d**) Apparent diffusion coefficient (ADC) map does not show a markedly hypointense signal corresponding to the lesion seen in (**c**). DWI PI-RADS = 3, ADC PI-RADS = 1, PI-RADS assessment category = 3.

Figure 4 shows the findings for one patient with a PI-RADS v2.1 score of 3. The patient's clinical parameters were as follows: age > 75 years, SII < 550, CysC level > 0.9, and PSAD > 0.25. Therefore, on the basis of the XYFY risk model, the patient was scored 168 and classified in the high-risk group. Pathological results confirmed positive biopsy results in this patient.

## Discussion

In this study, we used hematological and bpMRI parameters to establish and internally validated a risk scoring model for predicting the biopsy outcomes for patients with PSA levels of 4–20 ng/mL and this model would support clinical decision-making and reduce excessive biopsies.

PSA is the most commonly used marker for the diagnosis of PCa. The serum PSA level between 4 ng/mL and 10 ng/mL was often referred to as the "gray zone"^21^. However, PSA levels and PCa prevalence were different among ethnic groups. For Asian populations, the prior definition of the “gray zone” does not seem accurate enough, and can lead to over-detection if only based on PSA abnormality^22^. Studies have shown no significant difference in cancer detection rates in patients with a PSA level of 10–20 ng/mL in comparison with patients with a PSA level of 4–10 ng/mL^23^. The incidence of PCa in China is lower than that in Western countries, and when the PSA level was 4 to 20 ng/mL, the detection rate of PCa is 25% or less^3^. In addition, studies have shown that different prostate biopsy methods may change the PSA gray zone range^24^. Therefore, some researchers believe that the "gray zone" of PSA in Asian men should be higher than the traditional "gray zone"^25,26^. Thus, a PSA level of 4–20 ng/mL was the level we used for our research. Similar to previous studies, in this study, we found that less than one third (30.96%) cases with PSA levels of 4–20 ng/mL were diagnosed with PCa.

Age is also one of the diagnostic indicators of PCa. The prevalence of PCa is associated with age: it was only 0.01% in those younger than 45 years, increased to 0.34% in those aged 45–59 years, and was 2.42% in those aged 60–74 years. Moreover, among men aged over 70 years, the prevalence of PCa was the highest in carcinomas of the urogenital system^2^. Cormio et al.^27^ and Radtke et al.^28^ both incorporated age into their models, which showed good predictive performance (AUC: 0.80–0.87). Consistent with the previous studies, our study demonstrated that age was an independent risk factor for PCa.

In 1992, Benson MC et al.^29^ combined PV with PSA, and first proposed the concept of PSAD, which can better reflect prostate damage. Subsequently, a number of studies showed the value of PSAD for the diagnosis of PCa. Li et al.^30^ established a radiomics nomogram for predicting csPCa in PI-RADS 3 lesions, and indicated that PSAD was an independent predictor and significantly improved the predictive efficiency of the radiomics score (AUC: 0.881 vs. 0.939). Another study divided 701 patients into three groups to construct and validate nomograms based on PSAD and PI-RADS. It showed that the nomogram had good discrimination for PCa (AUC = 0.804). Falagario et al.^31^ identified an optimal strategy for biopsy-naive patients, in which the cut-off value for PSAD was 0.2. This value was different from the value obtained in our study (0.25), and the difference may be attributed to the fact that Falagario emphasized the sensitivity of PSAD for predicting PCa. Similarly, according to our investigation, PSAD was an independent risk factor for predicting PCa, and it also improved the predictive efficiency of PI-RADS (AUC: 0.836 vs. 0.881; *P* < 0.001), which was consistent with Li's findings.

The PI-RADS v2.1 was implemented into clinical practice in 2019 to better standardize prostate MRI examinations and interpretation^7^. Although version 2.1 still considers the function of mpMRI/bpMRI as being controversial, a number of studies have shown the value of bpMRI for the diagnosis of PCa in biopsy-naive men. The studies by Tamada et al.^8^ and Zawaideh et al.^9^ demonstrated that bpMRI had equivalent PCa detection rates to mpMRI. Xu et al.^10^ evaluated the diagnostic efficiency of bpMRI and mpMRI for PCa, and their results indicated that the AUC of bpMRI and mpMRI were 0.790 (0.732–0.840) and 0.791 (0.733–0.841), respectively. No statistically significant difference between the two methods was found. Pan et al.^32^ defined a new parameter based on bpMRI, which was calculated from the T2WI and DWI scores and showed greater PCa discriminating power (AUC = 0.900). Taking together, bpMRI and mpMRI showed nearly the same performance in PCa diagnosis. Moreover, in comparison with mpMRI, bpMRI serves as a quicker, less expensive, and less invasive screening tool for PCa^33,34^. Therefore, we collected bpMRI data according to PI-RADS v2.1. Multivariable analysis showed that PI-RADS v2.1 was an independent predictor of positive prostate biopsy findings (*P* < 0.05). PI-RADS v2.1 significantly improved the diagnostic performance of the nomogram (AUC: 0.832 vs. 0.916).

Inflammation, especially chronic inflammation, can lead to an increased risk of tumorigenesis, and approximately 20% of tumors are mainly caused by chronic inflammation^35^. Environmental exposure to bacterial and viral infections, predisposes the prostate to inflammation, with an elevated expression of inflammatory cytokines. These inflammatory cells and cytokines constitute the tumor microenvironment which promote tumourigenesis and progression by navigating the immunoregulatory network, phenotypic epithelial-mesenchymal transition (EMT), angiogenesis, and anoikis resistance^36^. While NLR and PLR are conventional inflammatory indicators^37–39^, SII, a new inflammatory index, appears to be a more powerful predictor of tumors than NLR and PLR^14,15^. Wang et al.^40^ found SII ≥ 471.86 (OR = 1.694; 95% CI 1.122–2.558; *P* = 0.003) was an independent risk factor and concluded that SII is the most powerful indicator among NLR, PLR, and SII in terms of their associations with PCa. Moreover, Rajwa et al.^41^ found that higher SII levels often predict adverse pathologic features (OR = 2.55; 95% CI 1.33–4.97; *P* = 0.005) and are a negative prognosticator of cancer-specific survival and overall survival. Therefore, we considered incorporating SII into our models. Similar to the findings of previous studies, our study revealed that SII was an independent predictor of biopsy outcomes (OR = 2.30; 95% CI 1.18–4.52; *P* = 0.015).

Maintenance of the balance between cathepsins and their inhibitors is important, but this balance can change in inflammatory diseases and malignancies^17,42^. CysC, a secreted cysteine protease inhibitor, is a potent inhibitor of cathepsin B(CTSB) and other lysosomal cysteine proteases^43^. It suggests that there may be a crosstalk between CysC and androgen receptor (AR)-mediated pathways. The low expression of CysC in prostate cancer enhanced the proteolytic activity of cysteine protease^17^. Previous study showed that the effect of CysC on modulating the prostate cancer cell invasion was provoked by crosstalk between cystatin C and AR-mediated pathways and Erk2 inhibitor that specifically inhibited MAPK/Erk2 activity^44^. CysC is widely expressed in the male reproductive system, and the concentration of CysC in benign prostate tissue is higher than that in other human tissues^45^. However, CysC can enter the blood due to malignant transformation of prostate tissue and cell destruction, so the peripheral blood CysC level in PCa patients is higher than that in normal patients. Yan et al.^46^ found that higher levels of CysC/CTSB in patients with esophageal cancer may be associated with longer overall survival (OS) (RR = 2.41; *P* = 0.001). In addition, CysC was inversely associated with PCa risk (HR per 1 standard deviation increase 0.93, 0.89–0.97, *P* = 0.001)^47^. Similar conclusions were obtained in our study, with multivariable analysis suggesting that CysC was an independent predictor for the outcomes of prostate biopsy (OR = 3.26; 95% CI 1.70–6.25; *P* = 0.001).

Biopsy is the gold standard for PCa diagnosis. However, unnecessary and excessive prostate biopsy is common. Several scholars have studied the value of hematological indicators, such as SII and CysC, for the diagnosis and combined diagnosis of PCa. Consistent with previous studies, we found that combining the PSAD with PI-RADS can improved the diagnostic efficiency of PI-RADS^30,31^. Based on the PI-RADS + PSAD model, we have added SII and CysC, which further improved the diagnostic value of the model. Our XYFY model has higher diagnostic efficiency and makes full use of the imaging and hematological indicators before biopsy compared to the Chinese Prostate Cancer Consortium Risk Calculator (CPCC-RC)^48^ and the model 6^49^. In addition, we transformed continuous variables into categorical variables, which was more convenient for clinicians to make decisions.

Several limitations of this study require consideration. First and foremost are the limitations inherent to its retrospective nature. In the second place, it is a single center study and further external validation is warranted. Thirdly, in this study, we enrolled patients between March 2010 and April 2021. During this period, the Gleason grading system changed; in addition, pathological diagnoses were made by different pathologists and the specimens were not re-reviewed.

## Conclusions

In conclusion, using bpMRI and hematological parameters, we constructed and internally validated a risk model to predict the likelihood of PCa at the initial prostate biopsy for Chinese patients with PSA levels of 4–20 ng/mL. This model performed significantly better than PI-RADS v2.1 (AUC: 0.90 vs. 0.84), potentially improving the criteria for prostate biopsy and reducing excessive prostate biopsies.

## Supplementary Information

Supplementary Information 1.

## Data Availability

The data supporting the conclusions used and/or analyzed in this study are available from the corresponding author by request.

## References

[1] Siegel RL, Miller KD, Fuchs HE, Jemal A (2022). Cancer statistics, 2022. CA Cancer J. Clin..

[2] Chen W, Zheng R, Baade PD, Zhang S, Zeng H, Bray F, Jemal A, Yu XQ, He J (2016). Cancer statistics in China, 2015. CA Cancer J. Clin..

[3] Lin YR, Wei XH, Uhlman M, Lin XT, Wu SF, Diao PF, Xie HQ, Xie KJ, Tang P (2015). PSA density improves the rate of prostate cancer detection in Chinese men with a PSA between 2.5–10.0 ng ml (−1) and 10.1–20.0 ng ml (−1): A multicenter study. Asian J. Androl..

[4] Kohaar I, Petrovics G, Srivastava S (2019). A rich array of prostate cancer molecular biomarkers: Opportunities and challenges. Int. J. Mol. Sci..

[5] Eastham JA, Auffenberg GB, Barocas DA, Chou R, Crispino T, Davis JW, Eggener S, Horwitz EM, Kane CJ, Kirkby E (2022). Clinically localized prostate cancer: AUA/ASTRO guideline, part I: Introduction, risk assessment, staging, and risk-based management. J. Urol..

[6] Mottet N, van den Bergh R, Briers E, Van den Broeck T, Cumberbatch MG, De Santis M, Fanti S, Fossati N, Gandaglia G, Gillessen S (2021). EAU-EANM-ESTRO-ESUR-SIOG guidelines on prostate cancer-2020 update. Part 1: Screening, diagnosis, and local treatment with curative intent. Eur. Urol.

[7] Turkbey B, Rosenkrantz AB, Haider MA, Padhani AR, Villeirs G, Macura KJ, Tempany CM, Choyke PL, Cornud F, Margolis DJ (2019). Prostate imaging reporting and data system version 2.1: 2019 update of prostate imaging reporting and data system version 2. Eur. Urol..

[8] Tamada T, Kido A, Yamamoto A, Takeuchi M, Miyaji Y, Moriya T, Sone T (2021). Comparison of biparametric and multiparametric MRI for clinically significant prostate cancer detection With PI-RADS version 2.1. J. Magn. Reson. Imaging.

[9] Zawaideh JP, Sala E, Shaida N, Koo B, Warren AY, Carmisciano L, Saeb-Parsy K, Gnanapragasam VJ, Kastner C, Barrett T (2020). Diagnostic accuracy of biparametric versus multiparametric prostate MRI: Assessment of contrast benefit in clinical practice. Eur. Radiol..

[10] Xu L, Zhang G, Shi B, Liu Y, Zou T, Yan W, Xiao Y, Xue H, Feng F, Lei J (2019). Comparison of biparametric and multiparametric MRI in the diagnosis of prostate cancer. Cancer Imaging.

[11] Balkwill F, Mantovani A (2001). Inflammation and cancer: Back to Virchow?. Lancet.

[12] Zhao H, Li W, Le X, Li Z, Ge P (2020). Preoperative neutrophil-to-lymphocyte ratio was a predictor of overall survival in small renal cell carcinoma: An analysis of 384 consecutive patients. Biomed. Res. Int..

[13] Sonmez G, Tombul ST, Demirtas T, Demirtas A (2021). Clinical factors for predicting malignancy in patients with PSA < 10 ng/mL and PI-RADS 3 lesions. Asia Pac. J. Clin. Oncol..

[14] Chen JH, Zhai ET, Yuan YJ, Wu KM, Xu JB, Peng JJ, Chen CQ, He YL, Cai SR (2017). Systemic immune-inflammation index for predicting prognosis of colorectal cancer. World J. Gastroenterol..

[15] Huang H, Liu Q, Zhu L, Zhang Y, Lu X, Wu Y, Liu L (2019). Prognostic value of preoperative systemic immune-inflammation index in patients with cervical cancer. Sci. Rep..

[16] Montagnana M, Danese E (2016). Red cell distribution width and cancer. Ann. Transl. Med..

[17] Jiborn T, Abrahamson M, Gadaleanu V, Lundwall A, Bjartell A (2006). Aberrant expression of cystatin C in prostate cancer is associated with neuroendocrine differentiation. BJU Int..

[18] Gore JL, Shariat SF, Miles BJ, Kadmon D, Jiang N, Wheeler TM, Slawin KM (2001). Optimal combinations of systematic sextant and laterally directed biopsies for the detection of prostate cancer. J. Urol..

[19] Lee SJ, Oh YT, Jung DC, Cho NH, Choi YD, Park SY (2018). Combined analysis of biparametric MRI and prostate-specific antigen density: Role in the prebiopsy diagnosis of Gleason Score 7 or greater prostate cancer. AJR Am. J. Roentgenol..

[20] Hu B, Yang XR, Xu Y, Sun YF, Sun C, Guo W, Zhang X, Wang WM, Qiu SJ, Zhou J (2014). Systemic immune-inflammation index predicts prognosis of patients after curative resection for hepatocellular carcinoma. Clin. Cancer Res..

[21] Yoon DK, Park JY, Yoon S, Park MS, Moon DG, Lee JG, Schroder FH (2012). Can the prostate risk calculator based on Western population be applied to Asian population?. Prostate.

[22] Ferlay J, Shin HR, Bray F, Forman D, Mathers C, Parkin DM (2010). Estimates of worldwide burden of cancer in 2008: GLOBOCAN 2008. Int. J. Cancer.

[23] Chavan PR, Chavan SV, Chavan NR, Trivedi VD (2009). Detection rate of prostate cancer using prostate specific antigen in patients presenting with lower urinary tract symptoms: A retrospective study. J. Postgrad. Med..

[24] Sönmez G, Tombul T, Demirtaş T, Öztürk F, Demirtaş A (2019). A comparative study: Has MRI-guided fusion prostate biopsy changed the prostate-specific antigen gray-zone range?. Cureus.

[25] Tang P, Du W, Xie K, Deng X, Fu J, Chen H, Yang W (2013). Transition zone PSA density improves the prostate cancer detection rate both in PSA 4.0–10.0 and 10.1–20.0 ng/ml in Chinese men. Urol. Oncol..

[26] Chang TH, Lin WR, Tsai WK, Chiang PK, Chen M, Tseng JS, Chiu AW (2020). Zonal adjusted PSA density improves prostate cancer detection rates compared with PSA in Taiwanese males with PSA < 20 ng/ml. BMC Urol..

[27] Cormio L, Cindolo L, Troiano F, Marchioni M, Di Fino G, Mancini V, Falagario U, Selvaggio O, Sanguedolce F, Fortunato F (2018). Development and internal validation of novel nomograms based on benign prostatic obstruction-related parameters to predict the risk of prostate cancer at first prostate biopsy. Front. Oncol..

[28] Radtke JP, Wiesenfarth M, Kesch C, Freitag MT, Alt CD, Celik K, Distler F, Roth W, Wieczorek K, Stock C (2017). Combined clinical parameters and multiparametric magnetic resonance imaging for advanced risk modeling of prostate cancer-patient-tailored risk stratification can reduce unnecessary biopsies. Eur. Urol..

[29] Benson MC, Whang IS, Pantuck A, Ring K, Kaplan SA, Olsson CA, Cooner WH (1992). Prostate specific antigen density: A means of distinguishing benign prostatic hypertrophy and prostate cancer. J. Urol..

[30] Li T, Sun L, Li Q, Luo X, Luo M, Xie H, Wang P (2021). Development and validation of a radiomics nomogram for predicting clinically significant prostate cancer in PI-RADS 3 lesions. Front. Oncol..

[31] Falagario UG, Jambor I, Lantz A, Ettala O, Stabile A, Taimen P, Aronen HJ, Knaapila J, Perez IM, Gandaglia G (2021). Combined use of prostate-specific antigen density and magnetic resonance imaging for prostate biopsy decision planning: A retrospective multi-institutional study using the Prostate Magnetic Resonance Imaging Outcome Database (PROMOD). Eur. Urol. Oncol..

[32] Pan JF, Su R, Cao JZ, Zhao ZY, Ren DW, Ye SZ, Huang RD, Tao ZL, Yu CL, Jiang JH (2021). Modified predictive model and nomogram by incorporating prebiopsy biparametric magnetic resonance imaging with clinical indicators for prostate biopsy decision making. Front. Oncol..

[33] Jambor I, Verho J, Ettala O, Knaapila J, Taimen P, Syvanen KT, Kiviniemi A, Kahkonen E, Perez IM, Seppanen M (2019). Validation of IMPROD biparametric MRI in men with clinically suspected prostate cancer: A prospective multi-institutional trial. PLoS Med..

[34] Choi MH, Kim CK, Lee YJ, Jung SE (2019). Prebiopsy biparametric MRI for clinically significant prostate cancer detection with PI-RADS version 2: A multicenter study. AJR Am. J. Roentgenol..

[35] Hayashi T, Fujita K, Matsushita M, Nonomura N (2019). Main inflammatory cells and potentials of anti-inflammatory agents in prostate cancer. Cancers (Basel).

[36] Archer M, Dogra N, Kyprianou N (2020). Inflammation as a driver of prostate cancer metastasis and therapeutic resistance. Cancers (Basel).

[37] Sciarra A, Gentilucci A, Salciccia S, Pierella F, Del BF, Gentile V, Silvestri I, Cattarino S (2016). Prognostic value of inflammation in prostate cancer progression and response to therapeutic: A critical review. J. Inflamm. (Lond.).

[38] Man YN, Chen YF (2019). Systemic immune-inflammation index, serum albumin, and fibrinogen impact prognosis in castration-resistant prostate cancer patients treated with first-line docetaxel. Int. Urol. Nephrol..

[39] Sonmez G, Demirtas T, Tombul ST, Akgun H, Demirtas A (2021). Diagnostic efficiency of systemic immune-inflammation index in fusion prostate biopsy. Actas Urol. Esp. (Engl. Ed.).

[40] Wang S, Ji Y, Chen Y, Du P, Cao Y, Yang X, Ma J, Yu Z, Yang Y (2021). The values of systemic immune-inflammation index and neutrophil-lymphocyte ratio in the localized prostate cancer and benign prostate hyperplasia: A retrospective clinical study. Front. Oncol..

[41] Rajwa P, Schuettfort VM, Quhal F, Mori K, Katayama S, Laukhtina E, Pradere B, Motlagh RS, Mostafaei H, Grossmann NC (2021). Role of systemic immune-inflammation index in patients treated with salvage radical prostatectomy. World J. Urol..

[42] 赵浩, 葛鹏, 李望, 吴建强, 李子祥, 王军起. 术前血清胱抑素C水平对手术的肾癌患者预后的影响. 现代泌尿外科杂志 **25**(3):242–246,257 (2020).

[43] Friedrich B, Jung K, Lein M, Turk I, Rudolph B, Hampel G, Schnorr D, Loening SA (1999). Cathepsins B, H, L and cysteine protease inhibitors in malignant prostate cell lines, primary cultured prostatic cells and prostatic tissue. Eur. J. Cancer.

[44] Wegiel B, Jiborn T, Abrahamson M, Helczynski L, Otterbein L, Persson JL, Bjartell A (2009). Cystatin C is downregulated in prostate cancer and modulates invasion of prostate cancer cells via MAPK/Erk and androgen receptor pathways. PLoS ONE.

[45] Jiborn T, Abrahamson M, Wallin H, Malm J, Lundwall A, Gadaleanu V, Abrahamsson PA, Bjartell A (2004). Cystatin C is highly expressed in the human male reproductive system. J. Androl..

[46] Yan Y, Zhou K, Wang L, Wang F, Chen X, Fan Q (2017). Clinical significance of serum cathepsin B and cystatin C levels and their ratio in the prognosis of patients with esophageal cancer. Oncol. Targets Ther..

[47] Perez-Cornago A, Fensom GK, Andrews C, Watts EL, Allen NE, Martin RM, Van Hemelrijck M, Key TJ, Travis RC (2020). Examination of potential novel biochemical factors in relation to prostate cancer incidence and mortality in UK Biobank. Br. J. Cancer.

[48] Chen R, Xie L, Xue W, Ye Z, Ma L, Gao X, Ren S, Wang F, Zhao L, Xu C (2016). Development and external multicenter validation of Chinese Prostate Cancer Consortium prostate cancer risk calculator for initial prostate biopsy. Urol. Oncol..

[49] Lu YF, Zhang Q, Yao WG, Chen HY, Chen JY, Xu CC, Yu RS (2019). Optimizing prostate cancer accumulating model: Combined PI-RADS v2 with prostate specific antigen and its derivative data. Cancer Imaging.

