# Supplementary material for: Prebiopsy bpMRI and hematological parameter-based risk scoring model for predicting outcomes in biopsy-naive men with PSA 4–20 ng/mL
Source: Sci Rep. 2022 Dec 19;12:21895. doi: 10.1038/s41598-022-26242-7 (PMC9763436; doi:10.1038/s41598-022-26242-7)
Supplement: Supplementary file 1 — Supplementary Information 1. [file 41598_2022_26242_MOESM1_ESM.pdf]

Prebiopsy bpMRI and Hematological Parameter-Based Risk Scoring Model for Predicting Outcomes in Biopsy-Naive Men with PSA 4-20 ng/mL

Yuxin Zheng, Wang Li, Yang Zhang, Chi Zhang, Junqi Wang, Peng Ge

Department of Urology, the Affiliated Hospital of Xuzhou Medical University, Xuzhou, China.

[*]**Correspondence**: Peng Ge (gepengyzu@126.com)

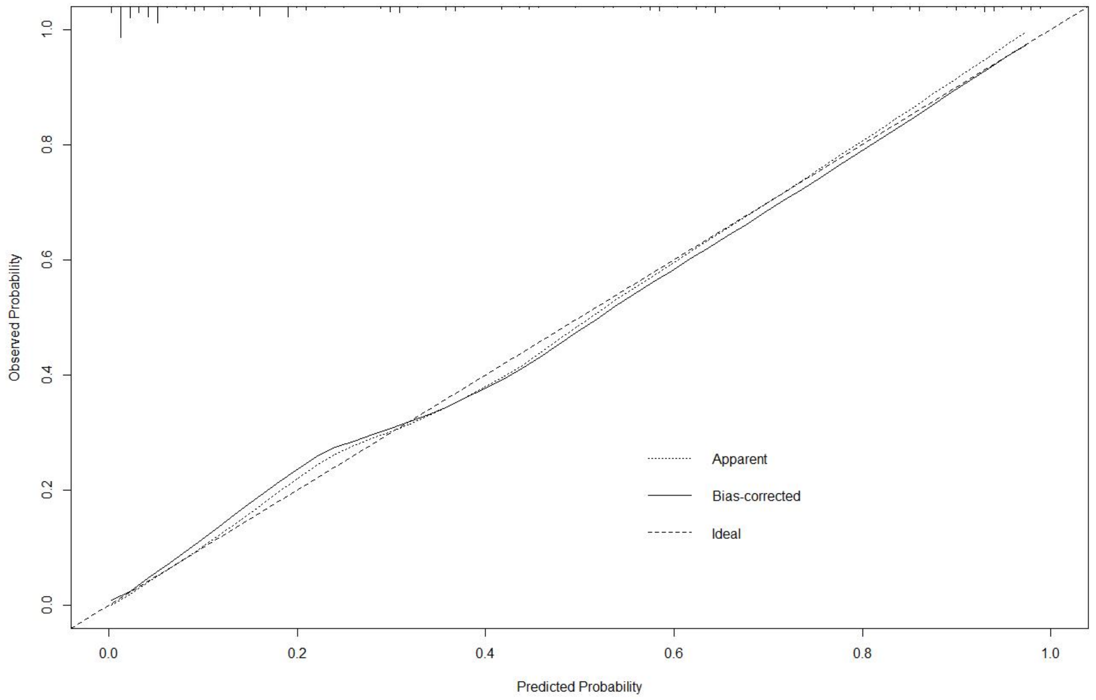

Figure S1. Calibration curve of the nomogram (1000 bootstrap resamples). The y-axis represents actual probability, and x-axis represents the probability estimated by nomogram. The apparent curve represents the relationship between predicted probability and actual probability. The bias-corrected curve was plotted by bootstrapping using 1000 resamples. The ideal curve means perfect prediction.

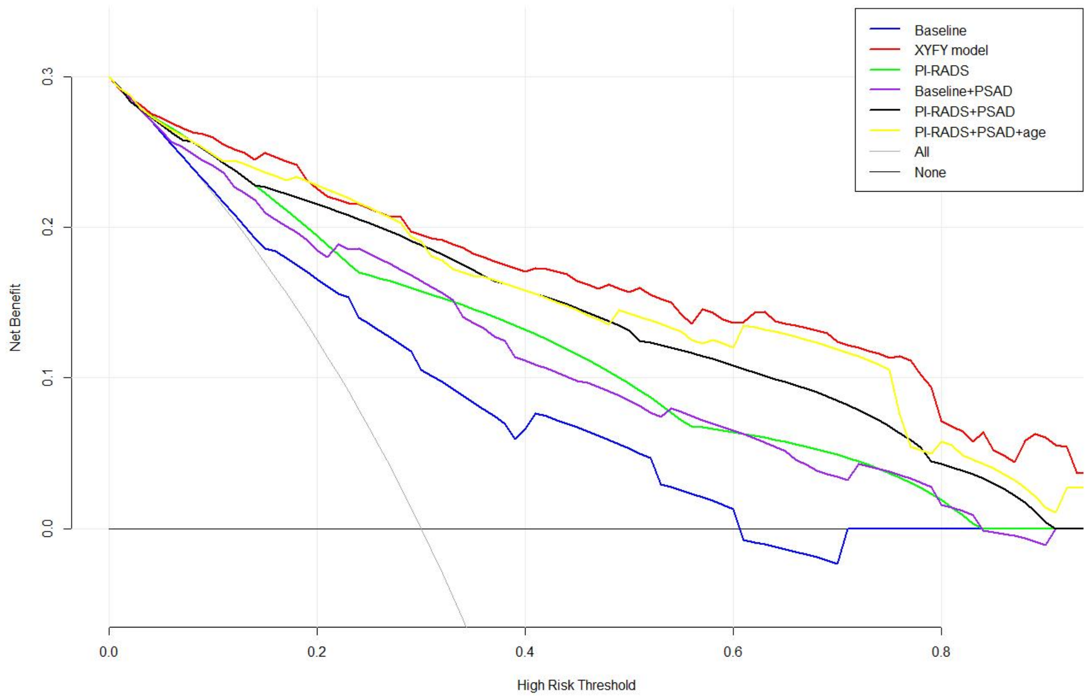

Figure S2. Decision curves analysis demonstrated the benefit of models for predicting the outcomes of biopsy. *PI-RADS* Prostate Imaging Reporting and Data System; *PSAD* prostate-specific antigen density.

Table S1. The results of PI-RADS v2.1.

Biopsy	PI-RADS v2.1 score			
	<3	3	4	5
Negative(n)	123	107	35	7
Positive(n)	4	35	46	37

PI-RADS v2.1 Prostate Imaging Reporting and Data System version 2.1.

Table S2. AUCs of the models.

Models	AUC	95%CI
PI-RADS	0.836	(0.794-0.878)
PI-RADS+PSAD	0.881	(0.845-0.918)
PI-RADS+PSAD+age	0.896	(0.862-0.930)
Baseline	0.745	(0.692-0.797)
Baseline + PSAD	0.832	(0.789-0.875)
XYFY	0.916	(0.886-0.947)

AUC area under the receiver operating characteristic curve; *CI* confidence interval; *PI-RADS* Prostate Imaging Reporting and Data System; *PSAD* prostate-specific antigen density.

Table S3. The detection of PCa and csPCa stratified by XYFY risk model and PI-RADS.

Histopathology	XYFY risk model			PI-RADS v2.1 score		
	<95	95-150	>150	<3	3	>3
Non-PCa	97.1% (195/201)	58.2% (71/122)	8.5% (6/71)	96.9% (123/127)	75.4% (107/142)	32.5% (40/123)
Pca	3.0% (6/201)	41.8% (51/122)	91.5% (65/71)	3.1% (4/127)	24.6% (35/142)	67.5% (83/123)
cisPCa	2.5% (5/201)	22.1% (27/122)	31.0% (22/71)	1.6% (2/127)	12.0% (17/142)	28.5% (35/123)
csPCa	0.5% (1/201)	19.7% (24/122)	60.6% (43/71)	1.6% (2/127)	12.7% (18/142)	39.0% (48/123)

csPCa clinically-significant prostate cancer; *cisPCa* clinically-insignificant prostate cancer; *PI-RADS v2.1* Prostate Imaging Reporting and Data System version 2.1.
